# Ensemble density-dependent synchronization of mycobacterial growth: BACTEC MGIT 960 fluorescence-based analysis and mathematical modelling of coupled biophysical and chemical processes

**DOI:** 10.3934/microbiol.2022017

**Published:** 2022-06-17

**Authors:** Anastasia I. Lavrova, Marine Z. Dogonadze, Alexander V. Sychev, Olga A. Manicheva, Eugene B. Postnikov

**Affiliations:** 1 Medical Department, St-Petersburg University, Universitetskaya emb 7-9, 199034, Saint-Petersburg, Russia; 2 St-Petersburg State Research Institute of Phthisiopulmonology, Lygovsky av. 2-4, 191036, Saint-Petersburg, Russia; 3 Research Center for Condensed Matter Physics, Kursk State University, Radishcheva st., 33, 305000 Kursk, Russia; 4 Department of Theoretical Physics, Kursk State University, Radishcheva st., 33, 305000 Kursk, Russia

**Keywords:** *M. tuberculosis*, synchronization, dense and dilute ensembles, kinetics, growth detection

## Abstract

This study presents an analysis of *M. tuberculosis* growth data obtained using the BACTEC MGIT 960 system and respective mathematical models. The system is based on the detection of a decrease in oxygen level in the broth due to the bacterial respiration. It is shown that recordings sampled with a 1 hour rate provide an opportunity to distinguish between the oxygen consumption of growing cells and active cells division when the density of micro-organisms is sufficient to enter into the synchronized division mode. More specifically, the growth of culture is continuous only with large initial dilutions; otherwise, there are jumps between different growth stages with a time interval of 13–15 h. The combination of the oxygen-quenching kinetics for an analytic reagent and the population growth kinetics resulted in a mathematical model, which consists of mixing Verhulst's and Gompertz's models. The parameters of such mixing and switching between the models' prevalences are discussed with respect to oxygen uptake reactions reflected in the changes in the experimentally registered fluorescence level.

## 1. Introduction

Biophysical methods focused on the rapid and accurate quantification of bacterial growth and its response to antibacterial drugs have recently attracted significant attention [1], [2]. Among these methods, the approaches addressing the optical indication of chemical reactions play an important role [3]–[5] because of their non-invasive character, reporting about biochemical content changes due to the metabolism of cells during their growth and proliferation. Therewith, such approaches take into account an amount of viable micro-organisms only that is impossible, say, for methods, which use the optical density measurements.

One of the most reliable processes for such studies is the bacterial respiration. The search for methods, which correlate the oxygen content in a medium, where bacteria grow, and characteristics of their vital activity was started at the dawn of quantitative experimental microbiology [6] and is continuing up to present, including such modern challenges as studying drug susceptibility and resistance [7]. Such an approach is based on two reasons: i) Oxygen consumption by breathing is a direct marker of different metabolic processes in living and dividing bacteria [8], [9]; ii) a dynamical change of the concentration of oxygen dissolved in a liquid medium can be accurately and effectively detected, registered and quantified by methods of analytical chemistry. It is possible due to the existence of a variety of neutral chemicals with photo-physical properties that drastically change in response to the content of oxygen dissolved in the medium under investigation. This approach finds applications to a wide range of systems: from aerodynamic flows and combustion problems to biological processes [10]–[12].

In particular, one of the most powerful methods, introduced about two decades ago [13]–[15] and widespread now as a standard in the field of investigations related to the bacterial cultures of *M. tuberculosis*, is the BACTEC MGIT 960 system. Although it operates with an indirect indication of the growth process, there exists a proportionality of the indicated growth units to the viable bacterial population; see e.g. [16], [17].

Its principle of operation can be summarized as follows: The Mycobacteria Growth Indicator Tube (MGIT) contains 7 mL of Middlebrook 7H9 medium supporting the growth of mycobacteria and silicon rubber impregnated with Tris(4,7-diphenyl-1,10-phenanthroline)ruthenium(II) dichloride complex, which is a fluorescence quenching based oxygen sensor. An activity of this fluorochrome is quenched by oxygen dissolved in the liquid broth. During the bacterial growth, the dissolved oxygen is consumed and, subsequantly, replaced with carbon dioxide. As a result, the fluorochrome inhibition by free oxygen gradually diminishes, and its fluorescence in response to the ultraviolet illuminations can be detected by the BACTEC's sensor. This procedure is carried out by the BACTEC MGIT 960 in an automated regime with the time step of one hour. However, the BACTEC MGIT 960 is used in conventional studies for a binary decision only: whether the fluorescence intensity overcomes some empirically prescribed boundary (this indicates a growing culture) or not (this is interpreted as a non-growing culture). In addition, the time required to cross the boundary mentioned can be taken into account.

At the same time, the BACTEC MGIT 960 can provide much more information, which can be analyzed and potentially may give important biophysical and biochemical insights, if one takes into account the rich data series with a fine time resolution. In particular, the promising target is the detailed study of *Mycobacterium tuberculosis* culture growth under conditions which lead to synchronous cell division. Note that this phenomenon for different bacteria, including *M. tuberculosis*, was detected by the counts of numbers of viable units (either cells or colonies) in earlier works [18], [19]. It exhibits itself as the sequential jumps in this quantity separated by the stable fixed time intervals that reflect simultaneous divisions. Later, it was discussed that the cell division is regulated by time but not the cell size [20], and the minimal doubling time is equal to 14.7 h in batch culture for the standard reference strain H37Rv [21].

The analysis of the full curve of the respiration-induced fluorescence supplied with “a marriage” of mathematical models of analytical chemistry addressing oxygen-quenched chemical reactions [12], [22], [23] and biophysical models of bacterial dynamics can unravel detailed vital processes of bacterial growth. Thus, the goal of our work is to understand when and under which conditions the synchronization regime occurs and how the bacterial population physiologically behaves within the inter-division time intervals. Another question is whether it is possible to reveal a correspondence and transitions between such regimes and typical bacterial growth curves (e.g., Verhulst's and Gompertz's) well-established for typical smooth population growth with saturation.

## 2. Materials and methods

All data on fluorescence curves, which will be discussed below, were obtained with BACTEC MGIT 960 (Becton Dickinson) for the case of the standard strain H37Rv of *M. tuberculosis* growing under conditions of the standard protocol [24], [25].

The initial inoculum further refereed to as “Dilution 1” is estimated as 7.5 × 10^6^ cells/mL. The control of the growth positivity corresponded to the instrument's indicator.

For further processing, the default output of the apparatus, which allows exporting recorded data in the format of Excel spreadsheets, was used. The program code operating with the default structure of such set-up-generated files for reading time series and their subsequent fitting can be found online at https://github.com/postnicov/readprocbactec

The general shape, magnitude, and time duration of the recordings do not contain any peculiarities. However, as was mentioned in the Introduction, the standard interpretation of such data is threshold-based and does not address the detailed time dynamics of these curves' shape evolution completely. On the contrary, the principal point of our interest and the main message of this section is “what new can be found and interpreted in the old-known curves when processing them with specially constructed methods.”

An additional test of the revealed dynamical peculiarities (see Discussion for details) was carried out using the resazurin reduction assay [26], [27], and quantitative growth curves were obtained photometrically using a portable colorimetric analyzer [28].

## 3. Results

### 3.1. Growth curves analysis

The BACTEC fluorescence growth curves, which indicate the growth of the bacterial population, look like a kind of logistic curves at large scales and are often not perfectly smooth, as they comprise periodically repeated concave sub-intervals. We hypothesize that such a behaviour is a possible direct consequence of synchronization processes, previous qualitative indication of which were mentioned above. Note that the high-frequency (with the time step of 1 hour) continuous monitoring of the oxygen-quenching fluorescence opens sufficiently wider opportunities for a more detailed study. To reveal this property precisely, we analyze not only the growth curves themselves but also their first and second derivatives (respectively, to the unit time step Δ*t* = 1 h), represented via the first

$$\Delta u(t_{n})=u(t_{n+1})-u(t_{n})\approx du/dt$$ (3.1)

and the second

$$\Delta^{2}u(t_{n})=u(t_{n-1})-2u(t_{n})+u(t_{n+1})\approx d^{2}u/dt^{2}$$ (3.2)

finite differences , which resemble the local growth speed and acceleration measured in g.u. · h^−1^ and g.u. · h^−2^, respectively.

**Figure 1.**
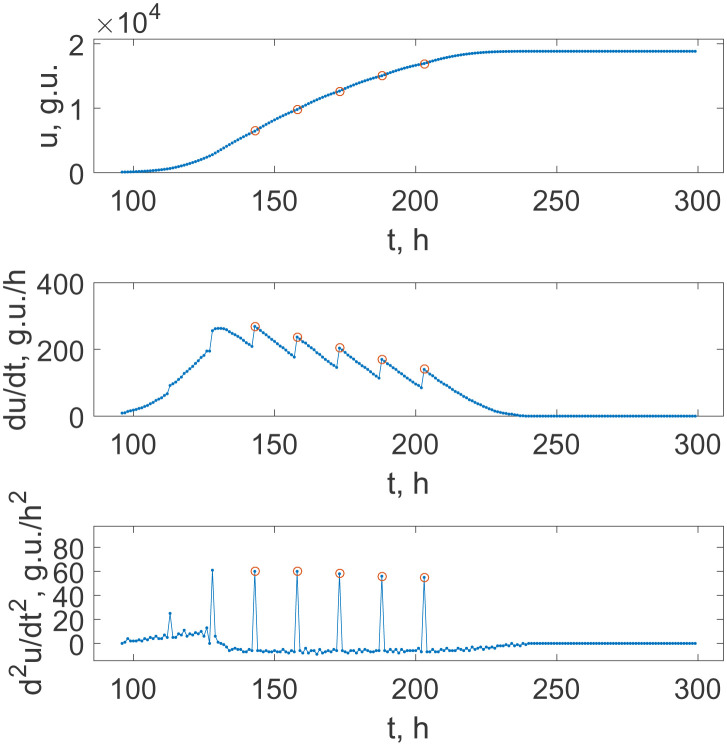
The time dependence of the fluorescence intensity indicating *M. tuberculosis* culture growth under the standard testing conditions (upper panel), and the first and the second derivatives of this growth curve (middle and lower panels, respectively), where g.u. means BACTEC's “growth units.” Dots show the recorded data with the equispaced 1 hour separation on the upper panel and the respective values obtained via the finite difference approximations of the derivatives; circles highlight breaking points of the monotonous growth used for the subsequent analysis; *t_b_* is a sequence of time moments at which the jumps occur, which are abscissas of the points denoted as circles.

**Figure 2.**
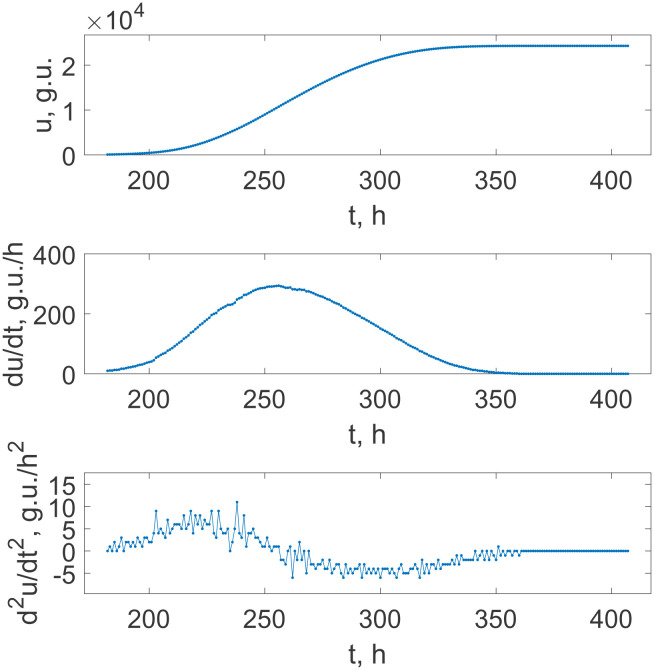
The time dependence of the fluorescence intensity indicating *M. tuberculosis* culture growth under the condition of 10^2^ times dilution of the initial culture seed (upper panel), and the first and the second derivatives of this growth curve (middle and lower panels, respectively). Dots show the recorded data with the equispaced 1 hour separation on the upper panel and the respective values obtained via the finite difference approximations of the derivatives.

**Figure 3.**
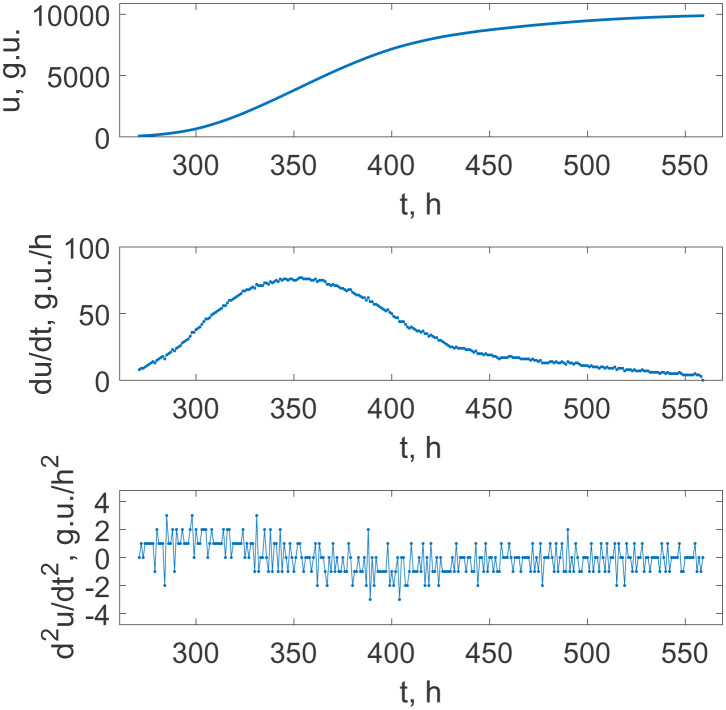
The time dependence of the fluorescence intensity indicating *M. tuberculosis* culture growth under the condition of 10^4^ times dilution of the initial culture seed (upper panel), and the first and the second derivatives of this growth curve (middle and lower panels, respectively). Dots show the recorded data with the equispaced 1 hour separation on the upper panel and the respective values obtained via the finite difference approximations of the derivatives.

Figures 1–3 illustrate this strategy for bacterial cultures prepared with different initial dilutions. Figure 1 relates to the case of the standard protocol of mycobacterial growth detection [25]. One can see that the growth is already non-monotonous, and, after some transient regime, the growth curve is subdivided into a stable periodic sequence of growth processes till the last stage leading to the saturated value.

Each periodically repeated stage is characterized by a jump-like initiation of the growth, which is followed by the practically linear decay of the growth speed, as is seen in Figure 1 (middle panel). The localization of these jumps can be easily performed by considering the second finite difference (3.2), which demonstrates a set of outliers, see Figure 1 (lower panel) where the corresponding points are highlighted by markers. The same markers subdivide the original BACTEC signal curve in Figure 1 (upper panel).

This procedure may be motivated as follows. The function *u*(*t*) is continuous but has salient points, where the derivative *du*/*dt* has breaks. Respectively, the derivatives of such discontinuous function *d*^2^*u*/*dt*^2^ = *d*/*dt*(*du*/*dt*) are equal to the Dirac delta-functions for the case of ideal continuous functions *u*(*t*) and sufficiently large values for the case of discrete samples. Since the experimental records *u*(*t_n_*) are sampled equispaced with respect to time, Eq (3.2) resembles such a second derivative.

One can see that the time distances between these outliers in the growth regime, which starts after some transient time and continues up to the last stage of the growth before the saturation, are quite stable and equal to *T_d_* = 15 hours for all curves in Figure 1. The absolute error of this quantity determination is Δ*T_d_* = 1 hours; that follows from the frequency of data measurements. Note that this value is quite close to the value of *M. tuberculosis* duplication time under conditions most favoured for the culture growth. Thus, we can conclude that this feature of the registered fluorescence curve argues in favour of the conclusion that bacteria in the culture are in the regime of synchronized growth and division.

Since synchronization as a rule requires a sufficiently dense packing of micro-organisms that makes available quorum sensing, the experiments were repeated with the initial seed culture under different degrees of dilution. Figure 2 demonstrates the growth situation for 10^2^ times diluted initial seed. One can see that these conditions result in a generally smooth curve, and the clearly expressed staged periodicity vanishes, which is confirmed by the plots of the first and second derivatives. Figure 2 (middle panel) does not demonstrate a saw-like pattern, and the data in Figure 2 (lower panel) do not exhibit significant point-wise jumps regularly exceeding those fluctuations along the whole curves, which are consequences of the non-smoothed finite difference procedure applied to the realistic record. Although some of the largest peaks in this plot of the second derivative may be leftovers of the sequential jumps, their magnitude does not significantly exceed the background noise (in contrast to Figure 1, where such a difference reaches almost two orders of magnitude). Therefore, it is not reasonable to explore them in detail.

The qualitatively same picture of a smooth growth is seen in the case of the 10^4^ times dilution of the initial seed culture, too; see Figure 3. Moreover, the plot of the second derivative in Figure 3 (lower panel) is more uniform in the magnitudes of fluctuations. They are associated with the equipment noise and leave no room for the staged growth process completely.

Thus, we can conclude that the emergence of periodic time-limited stages in the growth curve indicated by the overall fluorescence is connected with the density of the growing bacterial culture. The next step of the analysis was the quantifying these qualitatively distinct processes and associating them to the specificity of chemical reactions.

**Figure 4.**
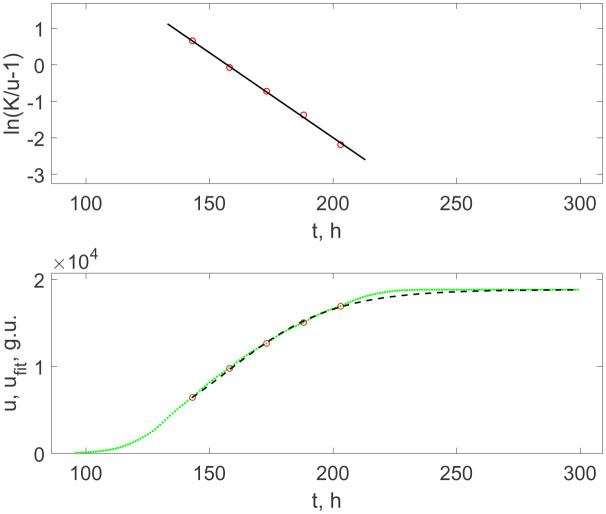
Upper panel: the linearized representation of the BACTEC's signal growth data taken at the transition points indicated in Figure 1 (circles) and their linear fitting, which corresponds to Verhulst's logistic growth function (solid line). Lower panel: the recorded data (green curve) as in the upper panel in Figure 1 and the Verhulst growth curve (dashed line) with the parameters determined by the fitting line shown in the upper panel.

First of all, let us consider the case corresponding to Figure 1. Due to the non-monotonous character of the growth curve *u*(*t*), where we identify breaking points of the first derivative localized at the time moments *t_b_* as the moments of synchronized cells divisions, we fit these points only at the first step. As it is shown in Figure 4 (upper panel), the points follow a straight line when the following special transformation is applied:

$$\text{ln}\left(\frac{K}{u(t_{b})}-1\right)=-r(t_{b}-t_{m}),$$ (3.3)

where *K* = max(*u*(*t*)) is the saturated value of complete sigmoidal growth curve, and *r* and *t_m_* are positive constants whose meanings will be explained below.

The dashed curve in Figure 4 (lower panel) shows the function

$$u_{fit}(t)=\frac{K}{1+exp\left(-r(t_{b}-t_{m})\right)}$$ (3.4)

obtained from Eq (3.3) and plotted as a continuous dependence on time but with the same parameters as in the discrete version (3.3). It is an explicit solution of the Verhulst equation, i.e., it defines the standard population growth limited by the carrying capacity of the environment with respect to the population size. Therefore, *r* is the population growth rate, and *t_m_* is the time of inflection, i.e., the moment when the population size reaches one half of the maximal (saturated) value *K*. The dashed curve (Figure 4, (lower panel)) accurately goes through the circles but deviates from the dotted curve denoting the fluorescent signal between and after them (and does not have sense before the first circle). Thus, the moments denoted by circles can be already associated with the population multiplication, while the remaining intervals originates from the specificity of chemical reactions governing the oxygen-quenched fluorescence that may be connected with the oxygen consumption by breathing bacteria.

**Figure 5.**
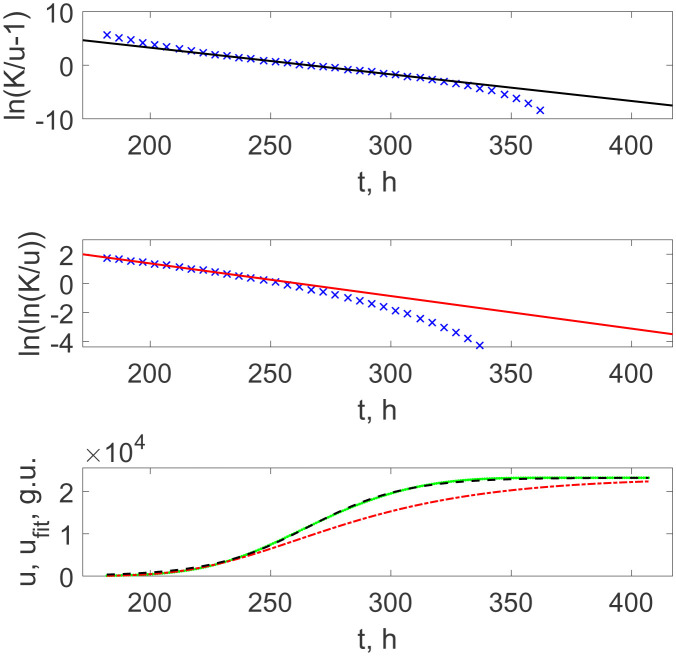
Upper panel: the linearized representation of the BACTEC growth curve for *M. tuberculosis* with 10^2^ times initial dilution (×'s separated by the time interval of 5 h for better visibility) and their linear fitting of its interval, which corresponds to Verhulst's growth function (solid line). Middle panel: the Fisher-Pry representation of the BACTEC growth curve for *M. tuberculosis* with 10^2^ times initial dilution (×'s separated by the time interval of 5 h for better visibility) and their linear fitting of its interval, which corresponds to Gompertz's growth function (solid line). Lower panel: the recorded data as in the upper panel in Figure 2 (dots), the Gompertz growth curve with the parameters determined by the fitting line shown in the upper panel (dash-dotted curve) and the Verhulst growth curve with the parameters determined by the fitting line shown in the middle panel (dashed curve).

The next case is the bacterial culture growth from 10^2^ times diluted seed. Figure 5 (upper panel) demonstrates the plot with the same representation by Eq (3.3) but now for the entire sequence of time moments *t* instead of a limited array of *t_b_*, since the curve is completely smooth, as shown in Figure 2. It should be noted that, for the sake of visibility, ×'s in Figure 5 are separated by the time intervals of 5 hours, but the actual computations did all use 1 hour-separated data points. One can see that that the middle part of the full curve in such representation follows the straight line. Moreover, the curve given by Eq (3.4) and shown as the dashed curve in Figure 5 (lower panel) practically coincides over the whole time region, and the deviation is small even in the initial and final regions, where the experimental markers in Figure 5 (upper panel) deviates from the fitting straight line.

At the same time, the data within the initial transient range can be approximated with another dependence in the form

$$\text{ln}\left[\text{ln}\left(\frac{K}{u(t)}\right)\right]=-r(t-t_{m})$$ (3.5)

(see Figure 5 (middle panel)), which defines Gompertz's sigmoidal growth

$$u_{fit}(t)=Ke^{e^{-r(t-t_{m})}},$$ (3.6)

which is shown as the dash-dotted line in Figure 5 (lower panel). Note that it reproduces the experimental recordings better than Verhulst's within the initial stage of growth only and then drastically deviates from the actual data. This can be explained by the fact that the cells in the initially diluted bacterial suspension are not synchronized with respect to their divisions yet. When this synchronization occurs, the growth dynamics changes to the logistic one, as for the cells' numerical multiplication points in Figure 4.

**Figure 6.**
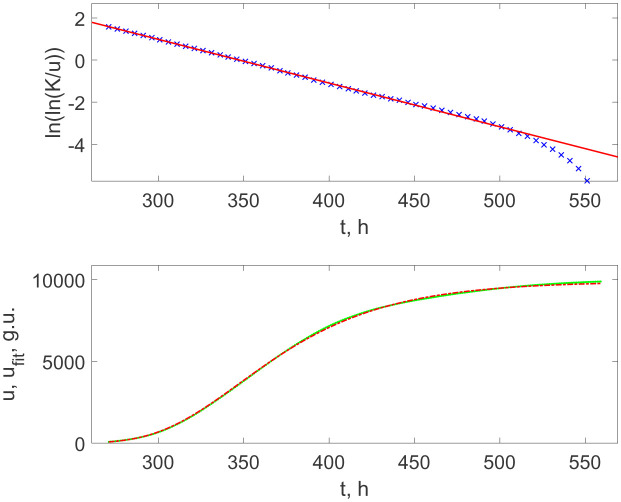
Upper panel: the linearized representation of the BACTEC growth curve of *M. tuberculosis* with 10^4^ times initial dilution (×'s separated by the time interval of 5 h for better visibility) and their linear fitting of its interval, which corresponds to Gompertz's growth function (solid line). Lower panel: the recorded data as in the upper panel in Figure 3 (dots) and the Gompertz growth curve with the parameters determined by the fitting line shown in the upper panel (dash-dotted curve).

However, when taking two orders more diluted initial suspension, then this asynchronous regime expands for more time, practically up to saturation; see the experimental data and their fitting by the function (3.5) in Figure 6 (upper panel). The respective analytical growth curve, the dash-dotted line in Figure 6 (lower panel), is practically indistinguishable from the recorded BACTEC's curve.

Table 1 lists the numerical parameters *r*, *t_m_*, and *K* included into Verhulst's and Gompertz's functions, (3.4) and (3.9), respectively.

One can see that both cases of the Verhulst growth curves, either with the clearly expressed jumps indicating the synchronization of divisions or without such jumps for more diluted culture, give the doubling times *T_d_* = ln/*r* equal, respectively, to *T_d_* = 14.9 h and 14.1 h, which completely agreed with the inter-jumps time intervals in Figure 1 equal to *T_d_* = (15 ± 1) h, taking into account the instrumental and fitting uncertainties. Thus, this confirms the conclusion that these jumps already correspond to the synchronized proliferation of bacteria.

On the other hand, the Gompertz's growth parameters revealed for the initial growth stage at the moderate (10^2^ times) dilution and for the full time of the process for the high (10^4^ times) dilution are also very close to each other. This result argues in favour of the conclusion that the Gompertz law of growth reflects the bacterial population's growth in the case when the culture density is too small to realize the synchronized duplication of *M. tuberculosis* in the whole community.

**Table 1.** The parameters of fitting sigmoidal curves shown in Figures 4–6.

Initial dilution	*r*/h^−1^	*t_m_*/h	*K*/g.u.
1	0.0466	157.1	18831
10^2^	0.049*	265.7*	23242
10^2^	0.022**	261.0**	23242
10^4^	0.021	347.3	9881

* for the Verhulst growth law component

** for the Gompertz growth law component

### 3.2. Possible kinetic mechanism

As is revealed by the analysis of growth curves in Figures 4–6, different dilutions of the initial seed culture leads to different growth laws. The high density of cells results in the growth curve (3.4), which resembles the logistic growth governed by the Verhulst equation

$$\frac{du}{dt}=ru\left(1-\frac{u}{K}\right)$$ (3.7)

if we consider time moments, when the fluorescence value corresponds to the moments of cell number multiplications.

At the same time, Eq (3.4) resembles the Stern-Volmer law for the fluorescence signal records under conditions of the oxygen-quenching kinetics

$$\frac{K}{u}=1+\alpha Q.$$ (3.8)

Here, we take into account that the signal *u* itself is the fluorescence signal recorded by the BACTEC system as a response to the diminishing oxygen quenching by oxygen dissolved in the medium. Comparing Eq (3.8) and Eq 3.4, we can conclude that the quenching oxygen density decays exponentially with time: *Q*(*t*) ~ exp(−*rt*). For the case of concentrated bacterial suspensions (Figure 4), this condition is fulfilled accurately at the points of the population's multiplication; for the less concentrated suspensions (Figure 6), it is the case for practically the whole time interval. Such a decaying dependence seems to be natural from the biophysical and chemical points of view, since vital bacteria already consume oxygen while breathing.

At the same time, Eq (3.7) implies that the growth rate of the multiplicative growth *du*/*dt* = *R*(*u*)*u* is limited, i.e., the per capita growth rate is a function *R* = *r*(1 – *u*/*K*) of the local cell density *u*, and stops when the local carrying capacity is reached. Thus, we can conclude that the growth is limited primarily by the organisms' packing reasons, not by the deficit of oxygen. This is also in accordance with the microbiology of *M. tuberculosis*, which are well-known as bacteria actively forming colonies and biofilms. In the case of a dense placement of such colonies formed by actively breathing newborn growing (but not yet dividing) cells in sediment in the bottom part of the tube, in the vicinity of the fluorescent sensor, they formed an oxygen-impoverished surrounding medium. This part induces the inter-division fluorescence level higher than Verhulst's growth curve. At the same time, the oxygen consumption rate decays when cells are maturing and synchronizing due to cells being born at the same time; see Figure 1 (middle panel). Therefore, oxygen from the upper part of the tube can diffuse to its bottom and to damp the extra fluorescence. Note that a similar concurrence of processes is revealed recently for some other model chemical reactions when characteristic reaction and diffusion times are comparable [29].

On the contrary, the initially highly diluted culture may not form such densely packed agglomerations such that this plays the role of a primary growth limiting factor. The growth process takes sufficiently longer time (see Figure 6) i.e., the amount of the consumed oxygen is larger. Finally, there are no indications of any division synchronization processes. As a consequence, we can consider the population (and the fluorescence growth curve) as reflecting a growth process averaged over the whole sample *du*/*dt* = *Ru* whose growth rate is governed by the concentration *s* of dissolved oxygen *R* = *R*(*s*) ∝ s. Taking the latter as exponentially decaying, the resulting system of equations takes the form

$$\frac{du}{dt}=su,$$

$$\frac{ds}{dt}=-rs,$$

which can be represented as a single equation:

$$\frac{du}{dt}=-ru\text{ln}\frac{u}{K},$$ (3.9)

which is the Gompertz equation.

### 4. Discussion

Thus, our analysis showed that bacteria colonies may not grow smoothly but can exhibit a staged growth described by a curve with jumps in its derivative. However, this staged character disappears with dependence on the initial medium dilution. It should be noted that the time interval for cells' division can vary in the range 13–15 h. It corresponds to experimental data obtained for the strain of wild-type *Mycobacterium tuberculosis* H37Rv growing on Middlebrook 7H9 medium at 37 °C [30]. Although the authors used the monotonously growing fitting curve in this case, the exploration of their direct experimental data points clearly shows the existence of step-wise sequential growth patterns separated by around a 15 h interval. Note that the time resolution contains 3–4 dots per such time interval. At the same time, for the case of cold-sensitive mutants, some of which have longer duplication periods, there was revealed not only the sequence of several concentration curves concave upward with decaying DNA concentration growth rate between subsequent divisions but the synchronization of this process, too.

**Figure 7.**
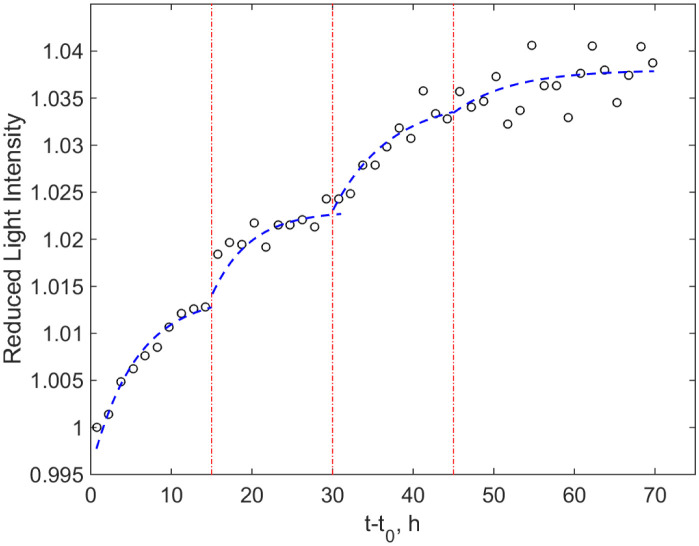
An example of the photometric curve indicating the growth of *M. tuberculosis* in a cell of the microbiological plate as a response of the color change due to the reduction of resazurin to resorufin compared to the control of pure resazurin. Markers indicate experimental data averaged over 1.5 h intervals (in middles of such intervals), and dashed curves are their fittings within subintervals denoted by vertical dash-dotted lines. For better visibility, the zero point of the time interval is shifted to demonstrate the region, where the change of resazurin color is detectable.

To make an additional test aimed at clarifying that the revealed staged growth curve is a feature of bacterial growth but not an instrumental artefact, we carried out an additional experiment using an alternative method for the growth registration: namely, the resazurin reduction assays. The reduction of blue resazurin to pink resorufin is connected with the respiratory activity of micro-organisms and belongs to the standard tests of their viability. The initial concentration of the pathogen suspension was the same as used for the experimental procedure in the BACTEC equipment (Dilution 1). The respective color change compared to pure resazurin (the control) was determined by applying the automated setup, registering the colour content by the transmitted light intensity. The details of the setup and principle of its operation can be found in the work [28]. The obtained experimental recordings are shown as markers in Figure 7; to highlight the interval of interest, the time scale origin is shifted in such a way that *t* = *t*_0_ corresponds to the beginning of the detectable colour change ≈ 3 days.

The time series of the data was not a smooth growth curve but a staged growth similar to Figure 1. To highlight this similarity to the data obtained by the BACTEC MGIT 960, the curves obtained by fitting the data to functions exponentially tending to saturation within subsequent 15 h subintervals are shown in Figure 7 with the dashed lines. Due to high scattering of photo-colorimetric data and limiting time resolution, it may not be logical to estimate a fitting coefficient. Figure 7 provides qualitative information and confirms that the staged growth with diminishing magnitudes of the growth rate's jumps detected with BACTEC MGIT 960 should be associated with peculiarities of the concentrated mycobacterial growing culture but not with the artefacts of the fluorescence registration, since the alternative method, not addressing fluorescence, exhibits similar behavior. There are a lot of phenomenological models of bacterial growth [31], but most used classical models, which generate sigmoidal curves resembling the great majority of data are Verhulst's and Gompertz's. In addition, they have direct interpretations from the point of view of chemical kinetics [32], mimicking a set of reactions, in which non only population density but also components of surrounding media can be included. However, it should be pointed out that more detailed study of such interactions [33] leads to the conclusion that a change of environmental conditions affects not only the growth rate but also other model parameters and even its type.

As for the possible effect of synchronization on the emergence of the staged growth, we note that it is known that one does not need to assume a perfectly synchronously dividing culture, which is definitely too strong of a condition compared to the considered experimental system. Rubinow's maturity-time/population growth time theory [34] demonstrated that such a kind of growth curve can be obtained (and detected for some micro-organisms different from *M. tuberculosis*) even if the growth rates and subsequent division times are probabilistically distributed. In this case, if the culture starts to grow at approximately at the same time (this condition is fulfilled by the procedure of sample preparation in our experiments), there is a small number of jumps in the growth curves, the amplitudes of which diminish due to desynchronizing. After this, the curve goes to the saturation smoothly, as is shown in the work [35], generalizing this model in the case of a saturated growth. It is practically the same behavior as revealed in our system.

It should be pointed out that some works [36]–[38], which analyzed the microscopic picture leading to the Gompertz growth law, argued that this kind of dynamics originates from stochasticity in the system of multiplying agents. This stochasticity can include a probabilistic distribution of the instant growth stages in the ensemble or arise from small fluctuations in growth rates. This property distinguishes it form the the Verhulst (logistic) growth, which can be considered as a strong averaged description of a well-mixed population growing under fixed environmental conditions.

Our results, which demonstrate the transition between these two kinds of growth curves emerging due to the system's dilution and visible extinction of features indicating a presence of the synchronization reflected in the oxygen quenching reaction in the medium, confirm these model conclusions by the direct experimental data.

### 5. Conclusion

Thus, we have analyzed chemical changes in medium obtained using the special system BACTEC MGIT 960. This system detects fluorescence based on the oxygen quenching sensor responding to the oxygen decrease due to active cell division. Our analysis allowed for revealing and detailed tracing biochemical processes accompanying the growth of a culture of *M. tuberculosis*.

We have shown that staged or continuous growth depends on the initial culture dilution which may be explained by the effect of quorum sensing. This means that there exists a certain level of a colony's density in a liquid broth, which leads to the synchronization of cellular metabolism processes responsible for inter-division mass growth and coordinated division itself. Moreover, the respective chemical response demonstrates the possibility of quantitative characterization of both processes with respect to oxygen consumption. This makes available the use of these data for the further building of similar biochemical models of bacterial metabolism with accurate timing. In particular, it is shown that the classic problem of choice between Verhulst's and Gompertz's models can be associated with the specific process, which provides a leading output during the stages of population vital activity and the synchronization rate between micro-organisms.

Finally, it should be pointed out that the metabolism of *M. tuberculosis* is not an exception from general biophysics and biochemistry of bacterial cultures. Therefore, the obtained results can be transferred to the studies of different microbiological cultures' growths with similar conditions.
